# One-year breakthrough SARS-CoV-2 infection and correlates of protection in fully vaccinated hematological patients

**DOI:** 10.1038/s41408-022-00778-3

**Published:** 2023-01-05

**Authors:** José Luis Piñana, Lourdes Vazquez, Marisa Calabuig, Lucia López-Corral, Gabriel Martin-Martin, Lucia Villalon, Gabriela Sanz-Linares, Venancio Conesa-Garcia, Andrés Sanchez-Salinas, Beatriz Gago, Ana Facal, Irene Risco-Gálvez, María T. Olave, Ildefonso Espigado, Javier Lopez-Jimenez, José Ángel Hernández-Rivas, Alejandro Avendaño-Pita, Ignacio Arroyo, Elena Ferrer, Irene García-Cadenas, Clara González-Santillana, Alicia Roldán-Pérez, Blanca Ferrer, Manuel Guerreiro, María Suarez-Lledó, Angela Camara, Diana Campos-Beltrán, David Navarro, Ángel Cedillo, Anna Sureda, Carlos Solano, Spanish Hematopoietic Stem Cell Transplantation and Cell Therapy Group (GETH-TC), Rodrigo Martino

**Affiliations:** 1 Hematology Department, Hospital Clínico Universitario de Valencia, Valencia, Spain; 2 Fundación INCLIVA, Instituto de Investigación Sanitaria Hospital Clínico Universitario de Valencia, Valencia, Spain; 3 Hematology Department, University Hospital of Salamanca (HUS/IBSAL), CIBERONC and Cancer Research Institute of Salamanca-IBMCC (USAL-CSIC), 37007 Salamanca, Spain; 4 Hematology Division, Hospital Universitario Fundación Alcorcón, Madrid, Spain; 5 Hematology Division, Institut Català Oncologia-Hospital Duran i Reynals, IDIBELL, Universitat de Barcelona, Barcelona, Spain; 6 Hematology Division, Hospital General universitario de Elche, Elche, Spain; 7 Hematology Division, Hospital Clínico Universitario Virgen de la Arrixaca, Murcia, Spain; 8 Hematology Division, Hospital Regional Universitario Carlos Haya, Malaga, Spain; 9 Hematology Division, Hospital universitario y politécnico La Fe, Valencia, Spain; 10 Hematology Division, Hospital Arnau de Vilanova, Valencia, Spain; 11 Hematology Division, Hospital Clínico Universitario Lozano Blesa, IIS Aragon, Zaragoza, Spain; 12 Hematology Division, Hospital Universitario Virgen Macarena, Sevilla, Spain; 13 Hematology Division, Hospital Ramon y Cajal, Madrid, Spain; 14 Hematology Division, Hospital Universitario Infanta Leonor, Madrid, Spain; 15 Hematology Division, Hospital de la Santa Creu i Sant Pau, Barcelona, Spain; 16 Hematology Division, Hospital de Fuenlabrada, Madrid, Spain; 17 Hematology división, Hospital Infanta Sofia, Madrid, Spain; 18 Hematology Division, Hospital Clinic de Barcelona, Barcelona, Spain; 19 Institute of Experimental and Clinical Pharmacology and Toxicology, Center for Brain, Behavior and Metabolism (CBBM), University of Lübeck, Lübeck, Germany; 20 Microbiology department, Hospital Clinico Universitario de Valencia, Valencia, Spain; 21 Department of Medicine, School of Medicine, University of Valencia, Valencia, Spain; 22 Hematopoietic Stem Cell Transplantation and Cell Therapy Group (GETH-TC) office, Madrid, Spain

**Keywords:** Medical research, Haematological diseases

## Abstract

The long-term clinical efficacy of SARS-CoV-2 vaccines according to antibody response in immunosuppressed patients such as hematological patients has been little explored. A prospective multicenter registry-based cohort study conducted from December 2020 to July 2022 by the Spanish Transplant and Cell Therapy group, was used to analyze the relationship of antibody response over time after full vaccination (at 3–6 weeks, 3, 6 and 12 months) (2 doses) and of booster doses with breakthrough SARS-CoV-2 infection in 1551 patients with hematological disorders. At a median follow-up of 388 days after complete immunization, 266 out of 1551 (17%) developed breakthrough SARS-CoV-2 infection at median of 86 days (range 7–391) after full vaccination. The cumulative incidence was 18% [95% confidence interval (C.I.), 16–20%]. Multivariate analysis identified higher incidence in chronic lymphocytic leukemia patients (29%) and with the use of corticosteroids (24.5%), whereas female sex (15.5%) and more than 1 year after last therapy (14%) were associated with a lower incidence (p < 0.05 for all comparisons). Median antibody titers at different time points were significantly lower in breakthrough cases than in non-cases. A serological titer cut-off of 250 BAU/mL was predictive of breakthrough infection and its severity. SARS-CoV-2 infection-related mortality was encouragingly low (1.9%) in our series. Our study describes the incidence of and risk factors for COVID-19 breakthrough infections during the initial vaccination and booster doses in the 2021 to mid-2022 period. The level of antibody titers at any time after 2-dose vaccination is strongly linked with protection against both breakthrough infection and severe disease, even with the Omicron SARS-CoV-2 variant.

## Introduction

Patients with hematological disorders (HD) are at high risk of severe coronavirus infectious disease 2019 (COVID-19) caused by the new coronavirus (SARS-CoV-2) [1]. The dramatic impact of COVID-19 on hematological patients was observed during the early phase of the pandemic, with mortality rates then exceeding 25% [2–7]. However, encouraging preliminary data suggest that SARS-CoV-2 vaccination has played a major role in reducing the severity of breakthrough COVID-19 in these immunocompromised patients, with mortality dropping to less than 10% during 2022 [8]. A recent retrospective case series supports the reduced mortality (12.4%) of breakthrough COVID-19 (mainly with the alfa variants of concern (VOC) after vaccination in hematological patients, but with high burden of hospitalization (66.4%) and intensive care unit (ICU) admission (21.3%) irrespective of the vaccine doses administered or serological response [9].

The first generation of SARS-CoV-2 vaccines failed to prevent community transmission of the virus, in particular the most recent Omicron VOC, but protection still remained high against severe COVID-19 [10]. In fact, it is expected that most HD patients worldwide will be infected several times with different SARS-CoV-2 VOC despite vaccination and boosters, and a significant number of them could still experience a severe course. The poor humoral response and impaired cellular immunity in HD patients contribute to a higher incidence of post-vaccination SARS-CoV-2 infection and its severity [11, 12]. To date, there is a lack of studies evaluating the kinetics of breakthrough SARS-CoV-2 infection in a prospective fashion as well as its severity in vaccinated HD patients. Thus, we still do not know to what extent these patients will develop breakthrough SARS-COV-2 infection after vaccination, their potential severity and the risk factors for these breakthrough infections.

In view of these gaps, the current study analyzes the incidence, characteristics, severity and clinical and immune factors associated with breakthrough SARS-CoV-2 infections through a prospective observational registry conducted by the Spanish Hematopoietic Stem Cell Transplantation and Cell Therapy Group (GETH-TC) aimed at monitoring the humoral response to SARS-CoV-2 vaccines through 1 year after complete vaccination in a large cohort of 1551 HD patients.

## Patients and methods

### Study population

In December 2020 the Infectious Complications Subcommittee (GRUCINI) of the GETH-TC in collaboration with the Spanish Society of Hematology and Hemotherapy (SEHH) launched a national prospective multicenter registry aimed at evaluating the humoral response for SARS-CoV-2 vaccination and its duration through one year after full vaccination (defined as two vaccine doses for RNA vaccines or one dose for adenoviral vector-based vaccines). Characteristics of this registry have been described in detail elsewhere [13, 14]. The registry included consecutive HD patients who were vaccinated against SARS-CoV-2 between December 30, 2020 to June 30, 2021 in 21 participating Spanish centers. In June 2021 a specific form for booster doses and breakthrough SARS-CoV-2 infection was implemented in the registry. Patients were followed as initially planned and monitored for the development of SARS-CoV-2 reactive IgG antibodies (SCoV2-R-A) and breakthrough SARS-CoV-2 infection at different time points (3–6 weeks, 3, 6, and 12 months) after the complete vaccination schedule. The status of all included patients was updated on July 28, 2022. All patients included in this registry gave their signed informed consent in accordance with the declaration of Helsinki. The local Research Ethical Committee of the Hospital Clínico Universitario of Valencia approved the registry and study protocol (reference code 35.21).

### Inclusion criteria, cohort selection and data check

As of July 28, 2022, the GETH-TC registry recruited 1783 patients with different hematological disorders who were fully vaccinated against SARS-CoV-2. With the aim of assessing (1) the incidence and severity of breakthrough SARS-CoV-2 infection at one year after full vaccination (2) the effect of serological response at different time points and (3) the effect of booster doses, the current study focused on patients with complete demographic and survival data. We excluded 232 patients from 3 centers, initially included with limited data (only affiliation data), that did not obtain the institutional approval for serological testing leaving 1551 hematological patients for final study analysis. At the time of this analysis none of the patients had received pre-exposure prophylaxis with monoclonal antibodies.

Out of 1551 HD patients, 1398 (90.1%), 1174 (76%), 1023 (66%) and 849 (55%) had available serological assessment at 3–6 weeks, 3 months, 6 months, and 12 months after full vaccination, respectively. Of these 1250 (89%), 1070 (91%), 900 (88%) and 820 (97%) had quantitative assessment in BAU/mL available, respectively, reflecting a high rate of missing serology data over time. To analyze whether there were relevant unknown biases which led to not having serological testing, we compared the clinical characteristics of patients and the survival of those with and without complete serological data at different time points and we did not find any significant differences (See supplementary Table S1), suggesting random reasons for missing serological data. Accordingly, we used a listwise deletion analysis to evaluate the effect of antibody titers on the risk of breakthrough infection at each pre-specified time point. A major reason for having missing serologies, from our personal experience and through our interactions with all participating physicians/centers, the most common reasons involved the patients’ and investigators’ exhaustion and/or the difficulties in scheduling serological tests outside the routine schedule for outpatient visits in long-term survivors, as well as the institutional refusal for serological monitoring through academic non-funded studies while the patient’s death before the scheduled serological test was a logical reason for missing serological follow-up.

### Technical considerations and Definitions

We assessed humoral response at the specified time points using serological ELISA or chemiluminescence immunoassay according to their availability at the microbiology services of each participating center. Antibody detection or seropositivity was defined as detectable SARS-CoV-2-reactive IgG antibodies (SCoV2-R-A) at any level above the lower limit of detection level for each test used. As recommended by the SEHH in vaccinated individuals, serological testing included the detection of IgG against both the nucleocapsid (N) and surface (S) proteins (anti-N and anti-S IgG, respectively) [15]. Antibody levels in patients with quantitative assessment were normalized according to the WHO standards and results were reported as SCoV2-R-A binding antibody units per milliliter (BAU/mL). Supplementary Table S2 summarizes the technical characteristics of the serological tests used and the normalization of antibody titers to BAU/mL according to WHO standards.

Complete vaccination (full primary immunization) schedules were defined as per marketing authorization at the time of study conduct and included two doses for full primary immunization (except COVID-19 Vaccine Janssen®). An additional dose after completion of full primary immunization was considered as a booster dose.

Pre-vaccination SARS-CoV-2 infection was defined as patients with prior history of PCR-proven COVID-19 and/or positive SARS-CoV-2 serostatus (IgG and/or IgM) before the first vaccine dose.

Breakthrough SARS-CoV-2 infection was defined as molecular (PCR test), antigenic or serological (IgG anti-N seroconversion between two consecutive serological tests) evidence of SARS-CoV-2 infection 7 days after full vaccination and until the last follow-up. Commercial PCR test used for diagnosis was provided in Supplementary Table S3. Corticosteroids use was defined as ≥15 mg per day of prednisone or equivalent doses of methylprednisolone, dexamethasone or hydrocortisone started at least 2 weeks before vaccination.

Although we did not sequence SARS-CoV-2 strains in any case, the inference of VOC on each episode was based on the likelihood of having a specific VOC according to the Spanish sequencing epidemiological data (see Supplementary Fig. S1) [16]. We inferred a specific VOC when the infection was diagnosed during the predominance (>50% of all sequenced VOC) of a specific VOC in our country. For example, Alfa-Beta VOC was considered in SARS-CoV-2 infections diagnosed from April 2021 to June 20 2021. Delta was considered from June 21 to December 26, 2021 and Omicron from December 27, 2021 to the end of the study in July 2022.

### Endpoints and statistical analysis

The primary objective of the study was to assess the incidence, severity and risk factors of breakthrough SARS-CoV-2 infection in vaccinated HD patients. Secondary endpoints include the effect of SCoV2-R-A titers on the development and severity of breakthrough SARS-CoV-2 infection at each pre-specified serological time points. We also evaluated the clinical benefit of booster vaccine doses as compared to those without boosters in terms of breakthrough infection incidence. Finally, we evaluated the effect of breakthrough SARS-CoV-2 infection in all-cause mortality.

The main patient characteristics were reported by descriptive statistics on the total available information: medians and ranges were used for continuous variables, while absolute and percentage frequencies were used for categorical variables. For comparisons, Fisher exact test or Mann–Whitney’s *U* test were used when appropriate.

Breakthrough SARS-CoV-2 infection after vaccination was estimated by the cumulative incidence method considering death without SARS-CoV-2 infection as the competing event [17, 18]. Univariate and multivariate analyses of risk factors for breakthrough infection were calculated using the Fine and Gray test [19]. For multivariate analysis, only variables with parameter estimates showing a *p* ≤ 0.10 in the univariate analysis were finally included. We did not include in the multivariate analysis time-dependent covariates (serological assessments and/or booster doses) since the circulation burden of SARS-CoV-2 in the community was not constant over time [20] which may lead to a misleading assumption (i.e. the booster vaccine doses were given after the second dose and just before the increase of community SARS-CoV-2 circulation and its inclusion as a time-depend covariate could lead to a misleading significant increase in the risk of breakthrough infection). To address the clinical benefit of booster doses as compared to those who only received 2 doses in terms of breakthrough infection incidence we performed two landmark analyses, the first one selecting a control group of patients (only 2 doses) alive without breakthrough infection and whose observation period started at the median time of booster administration in the study group (median time from second to third vaccine dose). The second landmark analysis started the observation period in December 27, 2021 in both groups (booster and non-booster) including patients alive and COVID-free at the beginning of the observation period to assure that booster and non-booster patients did not differ in the rate at which they came in contact with SARS-CoV-2 [21]. A median test analysis to check the protective effect of the amount of SCoV2-R-A was carried out in patients with available quantitative SCoV2-R-A titers normalized to BAU/mL, whether they developed or not breakthrough infections. A p-value <0.05 was considered statistically significant. All *p*-values are two-sided. Analyses were performed using the statistical software SPSS v. 25(IBM SPSS Statistics, Armonk, New York, USA) and R software, version 4.0.

## Results

### Patient characteristics

Patient characteristics are summarized in Table 1. Most patients (*n* = 1496, 96%) received complete vaccination with mRNA vaccines, and their median age was 63 years (range 16–97). Overall, the most common hematological diseases were B-cell non-Hodgkin’s lymphoma (B-cell NHL) (*n* = 260, 16.8%), plasma cell disorders (PCD) (*n* = 164, 10.6%), chronic lymphocytic leukemia (CLL) (*n* = 155, 10%) and chronic myeloproliferative neoplasms (cMPN) (*n* = 127, 8.2%). Among the cell therapy procedures, the most common was allo-HSCT (*n* = 429, 27.7%) followed by autologous stem cell transplantation (*n* = 121, 7.8%) and chimeric antigen receptor of T cell (CAR-T) therapy (*n* = 22, 1.4%). Pre-vaccine SARS-CoV-2 serologies were available in 514 patients (33%) and 29 of them (6%) were positive. Note that this series included 129 patients (8.3%) with prior evidence of SARS-CoV-2 infection before being vaccinated. Median follow-up after the second vaccine dose was 388 days (range 47–561), and for the 83% who received the 1st booster dose it was 189 days (range 0–302).

**Table 1:** Patient characteristics.

Characteristics	(*n* = 1551)
*Prior COVID-19, n (%)*	129 (8.3)
• Median time from COVID-19 to vaccination, days (range)	203 (11–422)
*Serological status prior to vaccination, n (%)*	
• Positive	64 (4)
• Negative	485 (31)
• Not tested	1002 (65)
*Type of 1st and/or 2nd vaccine dose, n (%)*	
• Moderna mRNA-1273	1086 (70)
• Pfizer-BioNTech BNT162b2	410 (26)
• Adenoviral vector-based	55 (4)
*Type of 3rd vaccine, n (%)*	1275 (82%)
• Moderna mRNA-1273	910 (71.3)
• Pfizer-BioNTech BNT162b2	361 (28)
• Adenoviral vector-based	4 (0.7)
*Age (years), median (range)*	63 (16–97)
• 18–40 years, *n* (%)	167 (11)
• 41–60 years, *n* (%)	551 (35.5)
• 61–70 years, *n* (%)	407 (26)
• >71 years, *n* (%)	426 (27.5)
*Male, n (%)*	871 (56.2)
*Baseline disease, n (%)*	
• AML	50 (3.2)
• ALL	5 (0.3)
• MDS	117 (7.5)
• B cell NHL	260 (16.8)
• T cell NHL	15 (1)
• Plasma cell disorders	164 (10.6)
• CLL	155 (10)
• HD	65 (4.2)
• cMPN	127 (8.2)
• Aplastic anemia	4 (0.3)
• Non-malignant disorders	17 (1)
• Allo-HSCT	429 (27.7)
• ASCT	121 (7.8)
• CAR-T	22 (1.4)
*Status disease at vaccination, n (%)*	
• Complete remission	823 (59.3)
• Partial remission	179 (11.5)
• Active disease	453 (29.2)
*Time last treatment to COVID-19 vaccine, months (range)*	
• Untreated	252 (16.2)
• Active treatment	441 (28.4)
• ≥6 months to 1 year	148 (9.5)
• ≥1 year	710 (45.8)
*Immunosuppressant drugs at vaccination, n (%)*	322 (20.8)
*Corticosteroids at vaccination, n (%)*	278 (17.9)
*Daratumumab, n (%)*	52 (3.4)
*Venetoclax, n (%)*	16 (1)
*Anti-CD-20 moAb, n (%)*	270 (17.4)
• <6 months before 1st vaccine dose	97 (6.3)
• 6 to 1 year before 1st vaccine dose	25 (1.6)
• >1 year before 1st vaccine dose	148 (9.5)
*BTK inhibitor therapy, n (%)*	67 (4.3)
*TKI therapy, n (%)*	49 (3.2)
*Lenalidomide maintenance, n (%)*	129 (8.3)
*Ruxolitinib therapy, n (%)*	15 (1)
*Blood count before vaccination (×10*^*9*^*/mL)*	
• Absolute neutrophile counts, median (range)	3.1 (0–46.7)
• Absolute lymphocyte counts, median (range)	1.73 (0.14–262.1)
• Absolute lymphocyte counts <1 × 10^9^/L	288 (18.6)
*Median time between 1st and 2nd vaccine doses, median days (range)*	28 (17–115)
*Time from 2nd dose to first serologies, median days (range)*	21 (12–62)
*Serological response at 3 weeks after 2 doses, n (%)*	
• Positive	1092 (70.4)
• negative	306 (19.7)
• Not available	153 (10)
*Third vaccine dose given, n (%)*	1284 (82.8)
*Time from 2nd dose to 3rd dose, days (range)*	168 (31–538)
*Median follow-up after 3 doses, days (range)*	189 (0–302)
*Serological response after 3 doses, n (%)*	
• Positive	251 (19)
• negative	72 (5.6)
• Not available	961 (75)
*Forth vaccine dose, n (%)*	430 (27.7)
*Time from 3rd dose to 4th dose, days (range)*	159 (31–266)
*Median follow-up after 4 doses, days (range)*	12 (0–95)
*SCoV2-R-A detection at 3–6 weeks after 2 vaccine doses, n/evaluable (%)*	1090/1398 (78)
• Antibody titers in BAU/mL, *n* (%)	1250 (89)
*SCoV2-R-A detection at 3 months after 2 vaccine doses, n (%)*	922/1174 (78)
• Antibody titers in BAU/mL, *n* (%)	1070 (91)
*SCoV2-R-A detection at 6 months after full vaccination, n (%)*	842/1023 (82)
• Antibody titers in BAU/mL, *n* (%)	900 (88)
*SCoV2-R-A detection at 1 year after full vaccination, n (%)*	740/849 (87)
• Antibody titers in BAU/mL, *n* (%)	820 (97)
*Breakthrough COVID-19, n (%)*	266 (17)
*Median time from 2 doses of vaccine to SARS-CoV-2 infection, days (range)*	86 (7–391)
*Median follow-up whole cohort, days (range)*	388 (47–561)
*All-cause mortality at median follow-up, n (%)*	89 (5.7)

*PCR* polimerase chain reaction, *AML* acute myeloid leukemia, *ALL* acute lymphoblastic leukemia, *MDS* myelodysplastic syndrome, *B-cell NHL* B-cell non-Hodgkin lymphoma, *T cell NHL* T cell non-hodgkin lymphoma, *CLL* chronic lymphocytic leukemia, *HD* Hodgkin disease, *MPN* chronic myeloproliferative neoplasm, *Allo-HSCT* allogeneic stem cell transplantation, *ASCT* autologous stem cell transplantation, *CAR-T* T-cell chimeric antigen receptor, *moAb* monoclonal antibody, *BTK inhibitor* Bruton’s tyrosine kinase inhibitor, *TKIs* tyrosine kinase inhibitors, *SCoV2-R-A* SARS-CoV-2-reactive IgG antibodies.

All patients were fully vaccinated, whereas 1284 (82.8%) received a first booster dose at a median of 168 days after complete vaccination schedule, and 430 (27.7%) received a second booster dose at a median of 159 days after the first booster.

### Incidence, severity, and risk factors of breakthrough SARS-CoV-2 infection

**Fig. 1:**
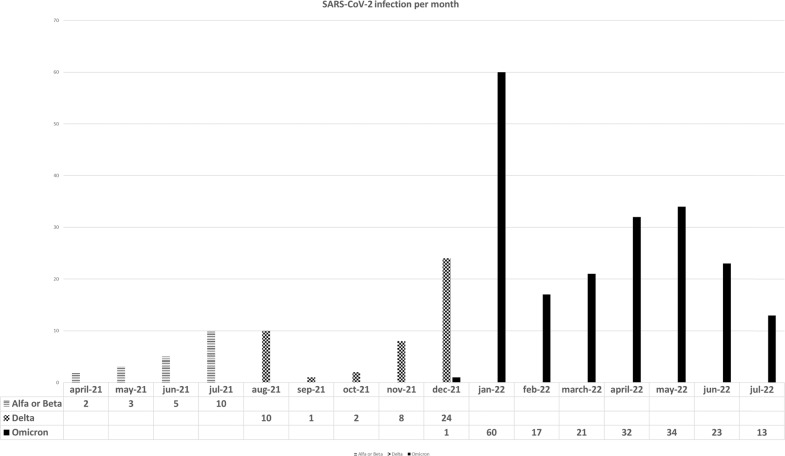
Epidemiological distribution of reported SARS-CoV-2 breakthrough infections in the whole cohort according to the likelihood of SARS-CoV-2 variants.

**Fig. 2:**
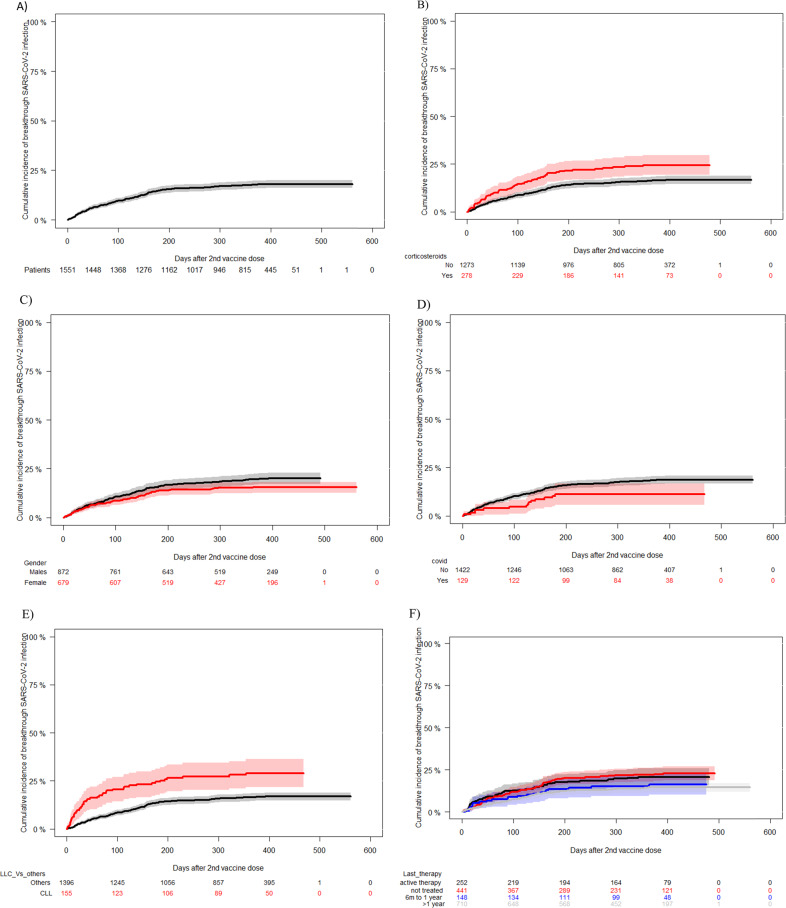
1-year Incidence of SARS-CoV-2 breakthrough infection. **A** In the whole cohort [cumulative incidence of 18% (95% confidence interval 95%, 16–20%)]. **B** According to the use of corticosteroids at the time of first vaccine dose. [Under corticosteroids cumulative incidence of 24.5% (95% confidence interval, 19–30%) vs without 16.7%, 95% C.I. 14–18 (*p* < 0.001)]. **C** According to the patient’s gender. [Male 19.6% (95% confidence interval, 17–22.4%) vs. female 15.5%, 95% C.I. 13–18 (*p* = 0.04)]. **D** According to development of COVID-19 before the first vaccine dose. [Prior COVID-19 11% (95% confidence interval, 6–17%) vs without 18.4%, 95% C.I. 16–20.5, (*p* = 0.04)]. **E** According to chronic lymphocytic leukemia vs others. [CLL 29% (95% confidence interval, 22–36%) vs. others16.8%, 95% C.I. 15–18.9 (*p* < 0.001)]. **F** According to timing of last therapy. [No treated 21% (C.I. 95%, 16–26%) vs. active therapy 20.5%, 95% C.I. 18–24 vs 6 months to 1 year 16% (C.I. 95%, 10–22) vs. >1 year 14% (95% C.I., 12–17) (*p* = 0.04)].

We identified 266 patients (17%) with breakthrough SARS-CoV-2 infection at a median of 158 days (range 7–391) after complete vaccination. Fourteen out of 266 (5%) episodes occurred in patients with pre-vaccination COVID-19 being detected by PCR (14/129, 11%). Characteristics of patients and SARS-CoV-2 breakthrough infection are summarized in Table 2. The distribution of breakthrough cases during the study period and type of probable VOC are provided in Fig. 1. Spanish epidemiological curve of SARS-CoV-2 infection during the study period was provided in Supplementary Fig. S2. The one-year cumulative incidence of breakthrough SARS-CoV-2 infection in the whole cohort from the start of vaccination was 18% [95% confidence interval (C.I.), 16–20] (Fig. 2A). Seventy-six (28.5%) developed SARS-CoV-2 infection after full primary vaccination and before the booster dose, whereas 165 (62% of breakthrough infections and 12.8% of those who received the 1st booster) and 25 (9.4% of breakthrough infections and 5.8% of those who received the 2nd booster) were infected after the 1st booster (before 2nd booster) and after the 2nd booster, respectively.

**Table 2:** Characteristics of patients with breakthrough SARS-CoV-2 infection.

Characteristics	SARS-CoV-2 infection (*n* = 266)
*Prior COVID-19, n/n evaluable (%)*	14/129 (10.8)
*Type of 1st and 2nd vaccines, n/n evaluable (%)*	
• Moderna mRNA-1273	189/1086 (17.4)
• Pfizer-BioNTech BNT162b2	68/410 (16.6)
• Adenoviral vector-based	9/55 (16.4)
*Type of 3rd vaccines before SARS-CoV-2 breakthrough, n evaluable (%)*	185 (69)
• Moderna mRNA-1273	133 (71.8)
• Pfizer-BioNTech BNT162b2	50 (27)
• Adenoviral vector-based	2 (1)
*Likely SARS-CoV-2 variants*^a^*, n (%)*	
• Alfa or Beta	18 (6.7)
• Delta	45 (17)
• Omicron	203 (76.3)
*Age (years), median (range)*	62 (19–97)
• 16–40 years, n/n evaluable (%)	32/167 (19.1)
• 41–60 years, n/n evaluable (%)	97/551 (17.6)
• 61–70 years, n/n evaluable (%)	60/407 (14.7)
• >71 years, n/n evaluable (%)	77/426 (18)
*Male, n (%)/n evaluable (%)*	165/871 (18.9)
*Baseline disease, n/n evaluable (%)*	
• AML	9/50 (18)
• ALL	0/5 (0)
• MDS	22/117 (18.8)
• B cell NHL	49/260 (18.8)
• T cell NHL	5/15 (33.3)
• Plasma cell disorders	32/164 (19.5)
• CLL	44/155 (26.8)
• HD	9/65 (13.8)
• cMPN	12/127 (9.4)
• Aplastic anemia	0/4 (0)
• Non-malignant disorders	5/17 (29)
• Allo-HSCT	53/429 (12.3)
• ASCT	23/121 (19)
• CAR-T	3/22 (13.6)
*Immunosuppressant drugs at vaccination, n /n evaluable (%)*	64/322 (19.8)
*Corticosteroids at vaccination, n /n evaluable (%)*	65/278 (23.3)
*Daratumumab, n /n evaluable (%)*	11/52 (21.1)
*Venetoclax, n /n evaluable (%)*	6/16 (37.5)
*Anti-CD-20 moAb, n /n evaluable (%)*	50/270 (18.5)
• <6 months before 1st vaccine dose	22/97 (22.6)
• 6 to 1 year before 1st vaccine dose	4/25 (16)
• >1 year before 1st vaccine dose	24/148 (16.2)
*BTK inhibitor therapy, n /n evaluable (%)*	17/67 (25.3)
*TKI therapy, n /n evaluable (%)*	12/49 (24.5)
*Lenalidomide, n /n evaluable (%)*	31/129 (24)
*Ruxolitinib therapy, n /n evaluable (%)*	1/15 (6)
***Timing and characteristics of breakthrough SARS-CoV-2 infection***	
*SARS-CoV-2 infection after 2 vaccine doses and before booster/s, n (%)*	76 (30)
• Median time, days (range)	158 (7–391)
*SARS-CoV-2 infection after 3 vaccine doses and before the 4th dose, n (%)*	165 (62)
• Median time, days (range)	121 (1–280)
*SARS-CoV-2 infection after 4 vaccine doses, n (%)*	25 (8)
• Median time, days (range)	24 (1–115)
*Number of evaluable SARS-CoV-2 infections after each serological time point, n (%)*	
• After 3–6 weeks from 2 doses	240 (90)
• After 3 months from 2 doses	207 (77.8)
• After 6 months from 2 doses	166 (62)
• After 1 year from 2 doses	37 (14)
*SCoV2-R-A detection at 3–6 weeks, n /n evaluable (%)*	170/240 (70.8)
*Median SCoV2-R-A titer at 3–6 weeks, BAU/mL (range)*	202 (0–10400)
*SCoV2-R-A detection at 3 months, n /n evaluable (%)*	137/200 (68.5)
*Median SCoV2-R-A titer at 3 months, BAU/mL (range)*	99 (0–15846)
*SCoV2-R-A detection at 6 months, n /n evaluable (%)*	120/161 (74)
*Median SCoV2-R-A titer at 6 months, BAU/mL (range)*	302 (0–48856)
*SCoV2-R-A detection at 1 year, n /n evaluable (%)*	27/37 (73)
*Median SCoV2-R-A titer at 1 year, BAU/mL (range)*	1017 (0–6423)
*Symptomatic SARS-CoV-2 infection, n /n evaluable (%)*	145/266 (54.5)
*Pneumonia, n /n evaluable (%)*	49/266 (18.4)
*Hospital admission, n /n evaluable (%)*	48/266 (18)
*Oxygen requirement, n /n evaluable (%)*	36/266 (13.5)
*ICU admission, n /n evaluable (%)*	5/266 (1.9)
*COVID-related Death, n /n evaluable (%)*	5/266 (1.9)
*All-cause mortality at median follow-up, n /n evaluable (%)*	12/266 (4.5)

*AML* acute myeloid leukemia, *ALL* acute lymphoblastic leukemia, *MDS* myelodysplastic syndrome, *B-cell NHL* B-cell non-Hodgkin lymphoma, *T cell NHL* T cell non-Hodgkin lymphoma, *CLL* chronic lymphocytic leukemia, *HD* Hodgkin disease, *MPN* chronic myeloproliferative neoplasm, *Allo-HSCT* allogeneic stem cell transplantation, *ASCT* autologous stem cell transplantation, *CAR-T* T-cell chimeric antigen receptor, *moAb* monoclonal antibody, *BTK inhibitor* Bruton’s tyrosine kinase inhibitor, *TKIs* tyrosine kinase inhibitors, *SCoV2-R-A* SARS-CoV-2-reactive IgG antibodies, *Anti-N* SARS-CoV-2 nucleocapsid antibodies, *ICU* intensive care unit.

^a^According to the Spanish epidemiological data regarding the predominance of each SARS-CoV-2 variant during the time of the study period, we considered Alfa or Beta VOC the episodes of SARS-CoV-2 infection diagnosed between April 1, 2021 and July 26, 2021, Delta VOC between July 27, 2021 and Omicron between December 27, 2021 to July 31, 2022.

Out of 266 cases, 145 (54.5%) had COVID-19 whereas 121 (45.5%) were asymptomatic infections. Pneumonia was documented in 49 cases (18.4%) whereas the SARS-CoV-2 infection-related hospital admission rate was 18% (*n* = 48). There were 5 COVID-19-related deaths (1.9%) at a median of 28 days (range 7–50) after SARS-CoV-2 detection.

Univariate and multivariate analyses of risk factors associated with the incidence of breakthrough SARS-CoV-2 infection are shown in Table 3. CLL patients (29%) and use of corticosteroids (24.5%) at the time of vaccination were associated with a higher cumulative incidence of breakthrough infection whereas female gender (15.5%), and vaccination at least 1 year after the end of HD therapy (14%) were protective in the multivariate analysis. As shown in Table 3, diagnosis of a cMPN and having had COVID-19 before vaccination showed a trend for a protective effect. The incidences of SARS-CoV-2 infection according to the aforementioned risk factors are shown in Fig. 2B–E. To analyze whether similar risk factors existed for breakthrough infection by the omicron variants, we performed a separate multivariate analysis in those infected by these VOC and found the same risk factors as in the entire cohort. We also performed a multivariate analysis of risk factor for symptomatic breakthrough SARS-CoV-2 infection and risk factors remain unchanged except for gender which was no longer significant (details of both analyses in the footnote to Table 3).

**Table 3:** Logistic regression univariate and multivariate analyses of factors predicting SARS-CoV-2 breakthrough infection after full vaccination.

	SARS-CoV-2 infection	*P*-Value	SARS-CoV-2 infection	*P*-value
Characteristics	Univariate Fine and Gray (95% C.I.)		Regression Fine and Gray^a^/^b^ HR (95% C.I.)	
*Prior to vaccination COVID-19*		0.04		
• Yes	11 (6–17)		0.6 (0.35–1.02)	0.06
• No	18.4 (16–2)			
*Type of vaccine*		0.5		
• Moderna mRNA-1273	17.9 (16–20)			
• Pfizer-BionTech BNT162b2	17.6 (14–21)			
• Adenoviral vector-based	18 (7–30)			
*Age (years)*		0.5		
• 18–40 years	20.7 (14–27)			
• 41–60 years	18 (15–22)			
• 61–70 years	15 (12–19)			
• >71 years	18.4 (15–22)			
*Sex*		0.067		
• Male	19.7 (15–20)			
• Female	15.5 (13–18)		0.75 (0.58–0.96)	0.025
*Baseline disease*		0.005		
• ALL	NT			
• AML	20 (8–32)			
• MDS	19.4 (12–27)			
• B cell NHL	20.2 (15–25)			
• T cell NHL	33 (9–57)			
• Plasma cell disorders	20 (14–27)			
• CLL	29 (20–34)		1.96 (1.37–2.79)	0.0002
• HD	14 (6–23)			
• cMPN	9 (3.9–14)		0.56 (0.31–1)	0.054
• Aplastic anemia or non-malignant disorders	28 (7–49)			
• Allo-HSCT	13 (10–16)			
• ASCT	21 (13–28)			
• CAR-T	14 (0–28)			
*Disease status at vaccination*		0.2		
• Complete remission	17.2 (15–19.7)			
• Partial remission	22.5 (16–29)			
• Active disease	17 (14–21)			
*Time from last treatment to COVID-19 vaccine*		0.004		
• Untreated	21 (16–26)			
• Under treatment	22 (18–26)			
• >6 months to 1 year	16 (10–22)			
• ≥1 year	14.5 (12–1)		0.92 (0.85–0.99)	0.04
*SCoV2-R-A at 3–6 weeks*		0.002	NT	
• Positive	16 (14–18)			
• Negative	25 (20–30)			
*SCoV2-R-A in BAU/mL at 3–6 weeks*		<0.0001	NT	
• >250	15 (12–18)			
• <250	25 (21–28)			
*Corticosteroids at vaccination*		0.001		
• Yes	24.5 (19–30)		1.41 (1.05–1.93)	0.02
• No	16.7 (15–19)			
*Daratumumab*		0.4		
• Yes	21 (10–33)			
• No	18 (16–20)			
*Venetoclax*		0.01	ns	
• Yes	38 (14–61)			
• No	17.9 (16–19.6)			
*Anti-CD-20 moAb*		0.5		
• Yes	19.5 (15–24)			
• No	17.5 (14–17.7)			
*Bruton’s TKI therapy*		0.07	ns	
• Yes	26 (15–37)			
• No	17.4 (16–19.7)			
*TKI therapy*		0.23		
• Yes	25 (13–37)			
• No	18 (16–19.8)			
*Lenalidomide*		0.02	ns	
• Yes	25 (17–33)			
• No	17.4 (15–19)			
*Ruxolitinib therapy*		0.3		
• Yes	7 (0–19)			
• No	18.2 (16–20)			
*Lymphocyte count* *<* *0.5 × 109/L*		0.5		
• Yes	16 (6–26)			
• No	18 (16–20)			
*Lymphocyte count* *<* *1.0 × 109/L*		0.3		
• Yes	20.6 (16–25)			
• No	17.4 (15–19.5)			

*AL* acute leukemia, *MDS* myelodysplastic syndrome, *B-cell NHL* B-cell non-Hodgkin lymphoma, *MM* multiple myeloma, *CLL* chronic lymphocytic leukemia, *HD* Hodgkin disease, *MPN* chronic myeloproliferative neoplasm, *Allo-HSCT* allogeneic stem cell transplantation, *ASCT* autologous stem cell transplantation, *moAb* monoclonal antibody, *TKIs* tyrosine kinase inhibitors, *SCoV2-R-A* SARS-CoV-2-reactive IgG antibodies.

^a^We carry out the multivariate analysis for Omicron VOC and found that risk factors remain unchanged:

Female gender HR 0.71 (C.I. 95%, 0.53–0.95), *p*-value 0.024.

LLC Vs others HR 2.01 (C.I. 95%, 1.33–3.04), *p*-value 0.0009.

Last therapy >1 year HR 0.88 (C.I. 95%, 0.77–1.00), *p*-value 0.051.

Corticosteroid use HR 1.681 (C.I. 95%, 1.16–2.41), *p*-value 0.005.

^b^Multivariate analysis for symptomatic breakthrough SARS-CoV-2 infection:

LLC Vs others HR 2.5 (C.I. 95%, 1.59–3.94), *p*-value 0.0007.

Last therapy >1 year HR 0.82 (C.I. 95%, 0.71–0.96), *p*-value 0.018.

Corticosteroid use HR 1.83 (C.I. 95%, 1.2 2.8), *p*-value 0.005.

### Antibody level titers and severity of breakthrough SARS-CoV-2 infection

**Fig. 3:**
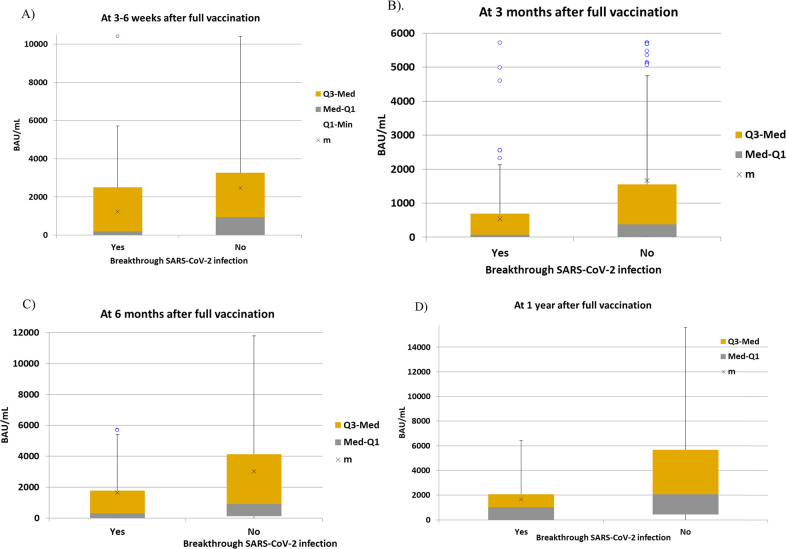
Median anti-SARS-CoV-2 IgG reactive antibodies titers measured in binding antibody units/mL (BAU/mL) at 3–6 weeks, 3 months, 6 months and 1 year after the 2nd dose according to the development of breakthrough SARS-CoV-2 infection. **A** Patients with breakthrough SARS-CoV-2 infection had a median of 202.57 BAU/mL (range 0–5714) vs those without SARS-CoV-2 infection median 937 BAU/mL (range 0–10400) (*p* < 0.001). **B** Q3-med means median interquartil 75%; Med-Q1, median interquartil 25%; m, mean. **C** Patients with breakthrough SARS-CoV-2 infection had a median of 70.14 BAU/mL (range 0–2126) vs those without SARS-CoV-2 infection median 379 BAU/mL (range 0–4746) (*p* < 0.001). **D** Patients with breakthrough SARS-CoV-2 infection had a median of 302 BAU/mL (range 0–5408) vs those with SARS-CoV-2 infection median 907 BAU/mL (range 0–11800) (*p* < 0.001). **E** Patients with breakthrough SARS-CoV-2 infection had a median of 1017 BAU/mL (range 0–6423) vs those without SARS-CoV-2 infection median 2080 (range 0–15600) (p < 0.001).

For this purpose, we excluded patients with breakthrough SARS-CoV-2 infection before each serological testing point. Median antibody titers according to the later development of breakthrough SARS-CoV-2 infection at different time points are shown in Table 4. As expected, the magnitude of SCoV2-R-A responses was significantly lower in patients with breakthrough SARS-CoV-2 infection as compared to those without at each of the serological points analyzed (Fig. 3A–D). Results were similar when analyzing cases attributed to the Omicron VOC (data not shown). Note that median antibody titers increased over time in both groups, reflecting the effect of booster doses, especially after 6 months from full vaccination. A serological cut-off value of 250 BAU/mL was able to discriminate the risk of being infected and the disease severity (Table 5).

**Table 4:** Median test analyses of the SARS-CoV-2-R-A titers in BAU/mL at different serological time point assessments according to occurrence of breakthrough SARS-CoV-2 infection^a^.

Variables	SARS-CoV-2 infection	No infection	*P*-value
*SARS-CoV-2-R-A at 3–6 weeks (n* *=* *1250)*	229	1021	
• Median Ab titers in BAU/mL (range)	202 (0–10400)	937 (0–56800)	<0.001
*SARS-CoV-2-R-A at 3 months (n* *=* *910)*	136	774	
• Median Ab titers in BAU/mL (range)	64.9 (0–5714)	380 (0–30460)	<0.001
*SARS-CoV-2-R-A at 6 months (n* *=* *841)*	144	697	
• Median Ab titers in BAU/mL (range)	302 (0–48842)	897.35 (0–95334)	<0.001
*SARS-CoV-2-R-A at 12 months (n* *=* 674)	36	638	
• Median Ab titers in BAU/mL (range)	1017 (0–6423)	2080 (0–87200)	0.003

*SARS-CoV-2-R-A* SARS-CoV-2 reactive antibodies, *Ab* antibodies.

^a^Results of median test remain unchanged when we only include evaluable cases for Omicron VOC.

**Table 5:** SARS-CoV-2 infection severity according to anti-SARS-CoV-2 IgG reactive antibody cut-off before the occurrence of SARS-CoV-2 infection in evaluable patients.

Variable	<250 BAU/mL At 3–6 weeks (*n* = 504)	≥250 BAU/mL At 3–6 weeks (*n* = 746)	*P* value	<250 BAU/mL At 3 months (*n* = 430)	≥250 BAU/mL At 3 months (*n* = 480)	*P* value	<250 BAU/mL At 6 months (*n* = 300)	≥250 BAU/mL At 6 months (*n* = 541)	*P* value
SARS-CoV-2 infection	119 (23.6%)	110 (14.7)	<0.001	86 (20%)	50 (10%)	<0.001	67 (22%)	77 (14%)	0.004
Symptomatic SARS-CoV-2	77/119 (65%)	45/110 (41%)	<0.001	56/86 (65%)	18/50 (36%)	0.001	43/67 (64%)	28/77 (36%)	0.001
Pneumonia	38/119 (32%)	7/110 (6%)	<0.001	21/86 (24%)	1/50 (2%)	<0.001	13/67 (19%)	3/77 (3.8%)	0.004
Hospital admission	38/119 (32%)	6/110 (5%)	<0.001	22/86 (25.5%)	0	<0.001	16/67 (23.8%)	3/77 (3.8%)	<0.001
Oxygen requirement	31/119 (33%)	3/110 (2.7%)	<0.001	19/86 (22%)	0	<0.001	13/67 (19%)	1/77 (1.2%)	<0.001
ICU admission	4/119 (3.8%)	1/110 (0.9%)	0.3	1/86 (1.2%)	0	0.9	0	1/77 (1.2%)	0.9
Death	5/119 (4.2%)	0	0.06	2/86 (2.4%)	0	0.5	3/67 (4.5%)	0	0.09

The median SCoV2-R-A levels at 3–6 weeks after full vaccination were markedly higher in asymptomatic breakthrough SARS-CoV-2 infection (*n* = 107) than in those with COVID-19 (*n* = 122) [870.67 BAU/mL (range 0–10400) vs 0 BAU/mL (range 0–5714.93), *p* < 0.001]. In addition, the median SCoV2-R-A levels were lower in those requiring hospital admission vs those who did not [0 BAU/mL (range 0–5456.88) vs 467.89 BAU/mL (range 0–10400), respectively, *p* < 0.001] and in those with pneumonia vs those without [0 BAU/mL (range 0–5456) vs 458 BAU/mL (range 0–10400), *p* < 0.001]. These findings were also true when we compared median SCoV2-R-A levels at 3 and 6 months after complete vaccination (data not shown).

### Infection severity, number of vaccine doses, and type of SARS-CoV-2 VOC

We analyzed potential differences in severity whether the patient had the breakthrough infection after the second, third, or fourth dose of vaccine (Table 6) and we did not find relevant differences, except for a trend to a lower rate of ICU admission after the third dose. In contrast, differences were more obvious according to the type of VOC (alfa-beta vs delta vs omicron) which was inferred from our National epidemiological sequencing data. Omicron causes milder cases of COVID-19 requiring less ICU admission than Alfa-Beta or Delta (*p* < 0.01 for both comparisons). There was a trend towards a lower pneumonia rate with Delta and Omicron. However, death occurred with Delta and Omicron but with very few cases (3/45 and 2/203, respectively).

**Table 6:** Severity of SARS-CoV-2 infection according to the likelihood of SARS-CoV-2 variant and the timing of the infection with regards to the vaccine dose.

Variable	After 2 doses (*n* = 81)	After 3 doses (*n* = 160)	After 4 doses (*n* = 25)	*P* value	Alfa or Beta (*n* = 18)	Delta (*n* = 45)	Omicron (*n* = 203)	*P* value
Symptomatic SARS-CoV-2	40 (49.5%)	90 (56%)	15 (60%)	0.5	8 (44%)	15 (33%)	122 (60%)	0.003
Pneumonia	19 (23%)	27 (17%)	3 (12%)	0.3	7 (38%)	8 (17.7%)	34 (16.7%)	0.06
Hospital admission	18 (22%)	28 (18%)	2 (8%)	0.26	6 (33%)	7 (15%)	35 (17%)	0.2
Oxygen requirement	11 (13%)	23 (14%)	2 (8%)	0.6	4 (22%)	5 (11%)	27 (13%)	0.48
ICU admission	4 (5%)	1 (0.6%)	0	0.051	2 (11%)	2 (4%)	1 (0.5%)	0.002
Death	2 (2.4%)	3 (1.8%)	0	0.7	0	3 (6.6%)	2 (1.9%)	0.03

### Booster doses and breakthrough SARS-CoV-2 infection

**Fig. 4:**
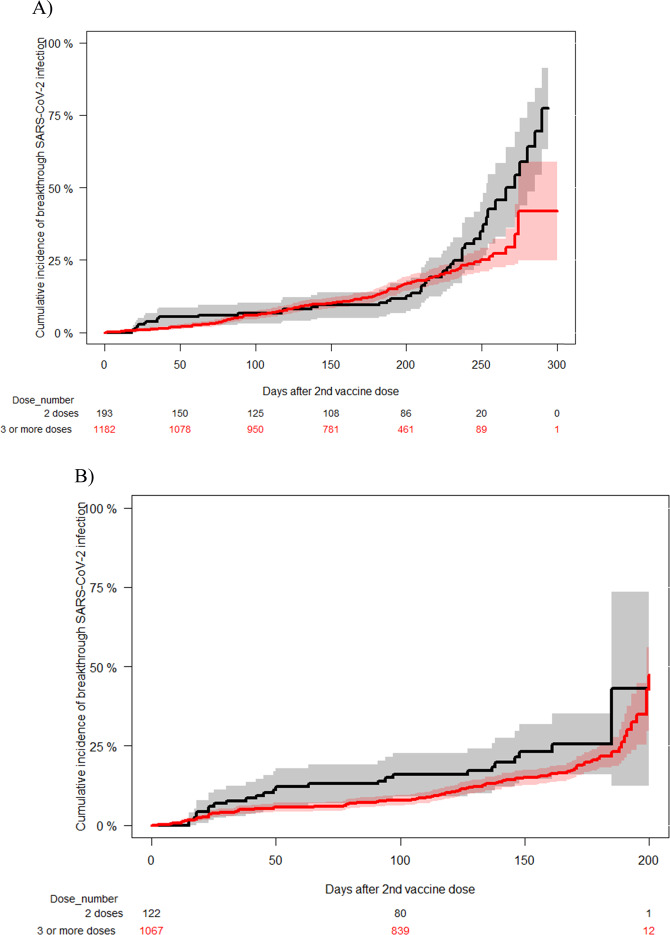
Landmark analyses of cumulative incidence of SARS-CoV-2 breakthrough infection according to the vaccine dose status. **A** Estimated from the time of third vaccine dose for booster cases and from median time between the 2nd and 3rd vaccine dose for the control group. [3rd dose 18% (95% confidence interval 16–21%) vs 2 doses 18%, 95% C.I. 12–25 (*p* = 0.1)]. **B** Estimated from December 26, 2021 in patients alive and without SARS-CoV-2 infection before the 3rd vaccine dose or before this date for the control group. [3rd dose 16% (95% confidence interval, 13–18%) vs 2 doses 23%, 95% C.I. 14–32 (*p* = 0.06)].

In order to evaluate the potential clinical benefit of receiving a booster dose, we performed a landmark analysis comparing the incidence of breakthrough SARS-CoV-2 infection in patients with or without a booster dose. The inclusion criterion in the booster group was no history of SARS-CoV-2 infection before the 1st booster dose (*n* = 1182), which was day 0 for the landmark analysis. The non-booster cohort included patients who were alive and COVID-19-free at the median time interval (plus 3 extra weeks) after the 2nd dose and the 1st booster dose in the booster cohort. There were no significant differences among groups regarding baseline diagnosis, corticosteroid use, time from last treatment to first COVID-19 vaccine or gender (data not shown). The cumulative incidence of breakthrough SARS-CoV-2 infection was similar among groups (booster group 18%, 95% C.I., 16–21 vs. non-booster group 18%, 95% C.I. 12–25, *p* = 0.1) (Fig. 4A). However, in this landmark analysis we observed that there was a progressive separation of both curves at the end of the observation period which coincided with a remarkable increase of SARS-CoV-2 circulation in the community (From December 26, 2021, see Figs. 1 and S2). To figure out if there was a significant difference among groups in the incidence of SARS-CoV-2 infection at the time of increased prevalence in the community we performed an additional landmark analysis where the observation period started on December 26, 2021 for both groups. For this analysis we included patients alive and without prior history of SARS-CoV-2 infection before that date and with at least 3 weeks of follow-up (*n* = 1067 in the booster group and *n* = 122 in the non-booster group). We observed a marginal benefit in the booster group (16%, 95% C.I., 13–18%) as compared to the non-booster group (23%, 95% C.I., 14–32) (*p* = 0.06) (Fig. 4B).

### Cause of death and all-cause mortality

Eighty-nine patients died (5.7%) in the whole cohort at a median of 233 days (range 48–442) after the first vaccine dose. Causes of death included progression or relapse of the underlying disease (*n* = 43, 48%), infections (*n* = 18, 20%), transplant-related complications (*n* = 9, 11%) and others (*n* = 19, 21%). Among those who developed breakthrough SARS-CoV-2 infection there were 12 deaths (4.5%) as compared to 77 (6%) deaths in those who did not (*p* = 0.35). Five patients (3 CLL and 2 NHL) died as a direct consequence of SARS-CoV-2 infection (1.9%). Three of them died after a third vaccine dose whereas 2 patients died after 2 doses.

## Discussion

The current study provides a real picture of SARS-CoV-2 breakthrough infections in patients with HD one year after the implementation of mass vaccination and boosters. The first observation was that the epidemiology of breakthrough SARS-CoV-2 infection in HD patients mirrored that of the community. Secondly, we identified conditions associated with higher incidence of breakthrough infection, such as CLL and corticosteroid use at the time of vaccination, whereas female gender and vaccination more than one year after the last treatment were associated with a reduced incidence. Quantitative humoral responses correlated with both the risk of developing breakthrough SARS-CoV-2 infection and its severity, irrespective of when it was evaluated. Unexpectedly, we observed an encouraging low mortality rate (1.9%) of breakthrough SARS-COV-2 infection in HD patients. In addition, breakthrough SARS-CoV-2 infections did not increase all-cause mortality in this large series of HD patients suggesting a favorable evolution of the pandemic in terms of virulence.

The prospective design of our registry along with the large sample size, the longitudinal serological monitoring and the long follow-up enable us to provide an accurate estimate of several critical issues, such as real-life epidemiology of SARS-CoV-2 infection in vaccinated HD patients and cumulative incidence estimates. In addition, our design limited the likely risk of bias of reporting only severe cases, which is characteristic of retrospective registry studies, providing a precise picture of clinical characteristics and severity of SARS-CoV-2 infection in this particular scenario.

Despite intensive transmission prevention measures counseling our patients and caregivers, we still observed that SARS-CoV-2 infection in hematological patients mirrored national epidemiological data [2, 20], since the three-peak prevalence of breakthrough SARS-CoV-2 infection observed in our series (July-August 2021, January 2022 and April-May 2022, see Fig. 1) fully coincided with our national epidemiological data (see Fig. 1 and Supplementary Fig. S2). Preventive transmission measures (hand washing, social distancing, wearing mask, etc) are still highly recommended for patients but are clearly far from being satisfactory. It looks suitable that such measures would be applied not only to immunosuppressed patients and their caregivers but also to those who have a close contact with these patients (friends, other relatives, health staff, etc). We already learned that the more thorough preventive transmission measures are, the lesser the incidence of respiratory virus infections in immunocompromised patients [22].

We report an overall cumulative incidence of breakthrough infection of 18% at one year after the start of mass vaccination. Although there is no available data in other scenarios for comparing cumulative incidence estimates, it is likely that HD patients have an increased breakthrough infection incidence as was the case in patients with solid tumors [23]. In fact, there is an evident increased risk of significant breakthrough infection immediately after vaccination in HD patients as compared to the general population [11], however, the incidence is not necessarily the same for all HD. Our findings suggest that CLL disease and receiving corticosteroid therapy at the time of vaccination experienced a significantly higher infection incidence. Several prior studies revealed that among HD patients, CLL patients are characterized by a severe cellular immune dysfunction which translates into a low humoral response rate [24, 25] and a severe course of the disease [26, 27], although a significant reduction in mortality has been observed in the Omicron era [28]. On the other hand, corticosteroid use has been associated with a lower probability of mounting an adequate humoral response [29]. Both conditions may facilitate the virus entry and in particular its spread. Although the use of anti-CD20 monoclonal antibodies has been consistently associated with a lower amount of SARS-CoV-2-R-A after vaccination [29–32], being treated with anti-CD20 monoclonal antibodies was not associated with an increased incidence of breakthrough infection in our study. It is likely that the number of events and in particular of patients who received anti-CD20 monoclonal antibodies within 6 months before starting vaccination (*n* = 97, 36%) was too low to observe significant differences. The male gender has been already associated with a more severe course of the disease [33]. In accordance, our multivariate analysis revealed that males were prone to higher incidence of breakthrough infection as compared to females. Although we do not have a likely explanation for this difference, we can speculate that hormonal differences and/or differences in social behaviors between males and females may explain such differences. Finally, patients free of treatment for at least one year before vaccination experienced a lower incidence, most likely because the probabilities of having a serological response after vaccination are consistently higher [29]. In light of these findings, it seems reasonable to maximize humoral vaccine responses according to the disease status and treatment as recently suggested by the SEHH [15] and that low-evidence-based pre-exposure prophylaxis recommendations in vaccinated patients with long-lasting monoclonal antibodies should be restricted to patients harboring these conditions instead of its unselective use. Of note, most of emergent Omicron BA. 4 and BA. 5 subvariants showed a loss of neutralization activity to almost all monoclonal antibodies but bebtelovimab [34].

Our prior preliminary analysis with only 37 cases of breakthrough infection, showed a higher rate of breakthrough infection and higher disease severity in patients without SARS-CoV-2-RA at 3–6 weeks of full vaccination [8]. With a significantly larger number of cases, we hereby confirm this observation and extended this finding beyond 3–6 weeks after full vaccination to later times points from vaccination and irrespective of the number of booster doses given. In this sense, higher levels of both binding and neutralizing anti-SARS-CoV-2 antibodies may protect against COVID-19 and are correlated with each other [35, 36]. Data in the general population also support this statement [37, 38]. Thus, the quantitative assessment of anti-SARS-CoV-2 antibodies at any time after vaccination could be justified for a benefit/risk calculation before additional vaccine doses or anti-SARS-CoV-2 monoclonal antibodies are given in these immunosuppressed population.

The severity of SARS-CoV-2 infection in patients with HD appeared to decrease over time most likely due to the implementation of mass vaccination programs. Initial reports, including ours, described high rates of pneumonia (>70%), hospital admission (>50%), ICU requirement (>20%) and overall mortalities exceeding 25% in unvaccinated HD patients [2–7]. A recent study focusing on breakthrough infections in vaccinated HD patients found a reduced severity but still a significant burden of hospitalizations (66%), ICU admissions (21.3%) and SARS-CoV-2-related mortality (12.4%) [9]. In contrast to this retrospective study, our data indicate that the severity is lower than previously reported in terms of hospitalizations (18%), ICU admissions (1.9%) and especially, mortality (1.9%) which was encouragingly low. These observations could be justified by several factors; first, the prospective design of the current study led to the capture of both symptomatic and asymptomatic infections, reducing the bias of registry-based retrospective studies. Second, the vast majority of cases (76%) were attributed to the Omicron VOC, which has a lower intrinsic virulence [28, 39]. Third, efforts in maintaining a high titers of anti-SARS-CoV-2 antibody titers in this vulnerable population may have paid off, as higher levels of antibody titers were consistently associated with lower/less disease severity. In fact, the cut-off of 250 BAU/mL was able to predict the risk of breakthrough infection as well as the disease severity.

Finally, we did not observe any significant differences according to the number of booster doses administered, which may merely indicate that more important than the number of booster doses is the amount of anti-SARS-CoV-2 antibody titers. In fact, no patient with SCoV2-R-A > 250 BAU/mL died from SARS-CoV-2 in our cohort irrespective of the serological time points assessed and the number of booster doses. The monitoring period during which our study was conducted (from early 2021 to end July 2022) spanned between the fifth, sixth and seventh COVID-19 waves in Spain, which were sequentially caused by Alfa-Beta, Delta and Omicron VOC according to our national epidemiological sequencing data. Current data show that infections by the Omicron VOC are more frequently symptomatic but less severe in term of ICU support compared to Alfa-Beta or Delta VOC. Our findings are in line with prior studies in the general population [39, 40].

The limitations of this study comprise the use of different serological tests, absence of neutralizing antibody testing, the absence of cellular immune response analyses and the lack of molecular data regarding the SARS-CoV-2 variants in patients with breakthrough infections. In addition, although a significant drop out at the 12 months after full vaccination period was observed and could be regarded as a limitation, all patients alive at the given follow-up time points were clinically assessed, and there were thus no patients who were alive and lost to follow-up during the study period. In this sense, the random appearance of serological missing data along with a still large number of evaluable cases and consistent results at each time point assessment may support a limited potential bias. The use of anti-N IgG seroconversion in defining breakthrough SARS-CoV-2 infection could be regarded as an additional limitation given the possibility of passive immunization transfer in patients receiving blood products support from seropositive donors. However, only 7.2% of patients with anti-N IgG seroconversion were on transfusion and/or immunoglobulin support at the time of vaccination.

## Conclusion

CLL patients and those under corticosteroids showed a higher incidence of breakthrough SARS-CoV-2 infection one year after the start of mass vaccination. The amount of antibody titers appears useful in identifying HD patients at higher risk of breakthrough SARS-CoV-2 infection by any VOC and its severity, and titers of SCoV2-R-A above 250 BAU/mL appear to be a good cut-off value. Last but not least, we report a reassuring reduction of SARS-CoV-2 mortality in this vulnerable population.

## Supplementary information

supplementary file

## Data Availability

Data available upon formal request by email to the Spanish hematopoietic transplant and cell therapy group (GETH-TC).
