# Supplementary material for: One-year breakthrough SARS-CoV-2 infection and correlates of protection in fully vaccinated hematological patients
Source: Blood Cancer J. 2023 Jan 5;13(1):8. doi: 10.1038/s41408-022-00778-3 (PMC9812742; doi:10.1038/s41408-022-00778-3)

**Table S1.** Clinical characteristics comparison according to the availability of complete serological data in BAU/mL through the study period.

| **Characteristics** | **Complete serological data (n= 820)** | **Incomplete serological data**  **(n=731)** | **P value** |
| --- | --- | --- | --- |
| **Prior COVID-19**, n (%) | 71 (8.7) | 58 (7.9) | 0.07 |
| **Serological status prior to vaccination, n (%)** |  |  | 0.34 |
| - Positive | 34 (10) | 30 (13) |  |
| - Negative | 290 (90) | 194 (87) |  |
| **Type of 1^st^ and/or 2^nd^ vaccine dose**, n (%) |  |  | 0.01 |
| - Moderna mRNA-1273 | 606 (73.9) | 480 (66) |  |
| - Pfizer-BioNTech BNT162b2 | 192 (23.4) | 218 (29.8) |  |
| - Adenoviral vector-based | 34 (4) | 21 (2.8) |  |
| **Type of 3^rd^ vaccine**, n (%) | 741 (90) | 534 (72) | 0.3 |
| - Moderna mRNA-1273 | 541 (73) | 369 (70) |  |
| - Pfizer-BioNTech BNT162b2 | 198 (26.8) | 163 (29.7) |  |
| - Adenoviral vector-based | 2 (0.2) | 2 (0.3) |  |
| **Age (years), median (range)** | 63 (18-97) | 62 (20-85) | 0.7 |
| - 18-40 years, n (%) | 82 (10) | 85 (11.6) |  |
| - 41-60 years, n (%) | 295 (36) | 256 (35) |  |
| - 61-70 years, n (%) | 221 (27) | 186 (25.4) |  |
| - >71 years, n (%) | 222 (27) | 204 (27.9) |  |
| **Male, n (%)** | 456 (55.6) | 415 (56.7) | 0.8 |
| **Baseline disease, n (%)** |  |  |  |
| - AML | 18 (2.1) | 32 (4) | 0.065 |
| - ALL | 2 (0.2) | 3 (0.4) |  |
| - MDS | 74 (9) | 43 (5.9) |  |
| - B cell NHL | 139 (16.9) | 121 (16.5) |  |
| - T cell NHL | 9 (1) | 6 (0.7) |  |
| - Plasma cell disorders | 83 (10.1) | 81 (11) |  |
| - CLL | 84 (10.2) | 71 (9.7) |  |
| - HD | 35 (4.2) | 30 (4.1) |  |
| - cMPN | 72 (8.7) | 55 (7.5) |  |
| - Aplastic anemia | 2 (0.2) | 2 (0.2) |  |
| - Non-malignant disorders | 11 (1.3) | 6 (0.8) |  |
| - Allo-HSCT | 219 (26.7) | 210 (28.7) |  |
| - ASCT | 59 (7.1) | 62 (8.4) |  |
| - CAR-T | 17 (2) | 5 (0.6) |  |
| **Status disease at vaccination, n (%)** |  |  | 0.2 |
| - Complete remission | 449 (54.7) | 374 (51.1) |  |
| - Partial remission | 86 (10.5) | 93 (12.7) |  |
| - Active disease | 241 (29.4) | 222 (30) |  |
| **Time last treatment to COVID-19 vaccine, months (range)** |  |  | 0.1 |
| - Untreated | 151 (18.5) | 101 (14) |  |
| - Active treatment | 217 (26.5) | 224 (30.5) |  |
| - ≥ 6 months to 1 year | 88 (10.7) | 60 (8.2) |  |
| - ≥ 1 year | 364 (44.3) | 346 (47.3) |  |
| **Immunosuppressant drugs at vaccination, n (%)** | 146 (18) | 176 (24) | 0.01 |
| **Corticosteroids at vaccination, n (%)** | 132 (16.1) | 146 (19.9) | 0.06 |
| **Daratumumab, n (%)** | 29 (3.5) | 23 (3.1) | 0.8 |
| **Venetoclax, n (%)** | 11 (1.3) | 5 (0.6) | 0.22 |
| **Anti-CD-20 moAb, n (%)** | 150 (18.3) | 120 (16.4) | 0.4 |
| - < 6months before 1^st^ vaccine dose | 46 (5.6) | 51 (6.9) |  |
| - 6 to 1 year before 1^st^ vaccine dose | 18 (2.1) | 7 (1) |  |
| - >1 year before 1^st^ vaccine dose | 85 (10.3) | 63 (8.6) |  |
| **BTK inhibitor therapy, n (%)** | 35 (4.2) | 32 (4.3) | 0.9 |
| **TKI therapy, n (%)** | 33 (4) | 16 (2.2) | 0.05 |
| **Lenalidomide maintenance, n (%)** | 54 (6.6) | 75 (10.2) | 0.01 |
| **Ruxolitinib therapy, n (%)** | 10 (1) | 5 (0.6) | 0.13 |
| **All-cause mortality at median follow-up,** n (%) | 9 (1) | 79 (10.8) | 0.001 |

**Table S2.** Characteristics of serological assays used in the study.

| Test | Manufacturer | Antibody target | SARS-CoV-2 antigen | BAU/mL |
| --- | --- | --- | --- | --- |
| - Architect SARS-CoV-2 IgG Quant II chemiluminescent microparticle immunoassay | (Abbott Diagnostics, Ill, USA) | IgG | RBD | AU x 0.142 |
| - Abbott ARCHITECT SARS-CoV-2 IgG | (Abbott Diagnostics, Ill, USA) | IgG | N |  |
| - Liaison SARS-CoV-2 S1/S2 IgG chemiluminescent assay | (DiaSorin S.p.A., Saluggia, Italy) | IgG | S1/S2 | AU x 2,6 |
| - MAGLUMI 2019-nCoV IgG chemiluminescent assay | (SNIBE—Shenzhen New Industries Biomedical Engineering Co., Ltd., Shenzhen, China) | IgG | S and N | AU/4.33 |
| - Elecsys anti-SARS-CoV-2 S | Roche Diagnostics (Pleasanton, CA, USA) | Total antibody (IgG, IgM, IgA) | RBD | U x 0.98 |
| - Elecsys® Anti-SARS-CoV-2 N | Roche Diagnostics (Pleasanton, CA, USA) | IgG | N |  |
| - Atellica SARS-CoV-2 | Siemmens <https://www.siemens-healthineers> (Germany) | Total IgG,IgM, IgA | RBD | U x 21.8 |
| S, Spike protein; N, nucleocapsid protein; RBD, receptor binding domain; AU, arbitrary units; U, units | | | |  |

**Table S3.** Commercial PCR test available in participating centers

| Alinity m SARS-CoV-2 Assay - Abbott Molecular (Ill, USA) |
| --- |
| Abbott RealTime SARS-CoV-2 Assay (Abbott (Ill, USA) |
| LightMix® Modular SARS-CoV (COVID-19) (Roche Diagnostics, Pleasanton, USA) |
| Aptima® SARS-CoV-2 Assay (Panther® System) (Hologic, Marlborough, Massachusetts, USA |
| TaqPath COVID-19 CE-IVD RT-PCR (Thermofisher Scientific Waltham, Massachusetts, USA |
| SARS-CoV-2 RT-PCR Vitro (Sevilla, Spain) |
| Xpert® Xpress SARS-CoV-2 Sunnyvale, California, USA |
| SARS-CoV-2 REAL TIME PCR KIT (Vircell, Granada, Spain) |

**Figure S1.** Spanish SARS-CoV-2 VOC sequencing epidemiological data.

Available at site: <https://www.sanidad.gob.es/profesionales/saludPublica/ccayes/alertasActual/nCov/documentos/COVID19_Actualizacion_variantes_20220523.pdf>


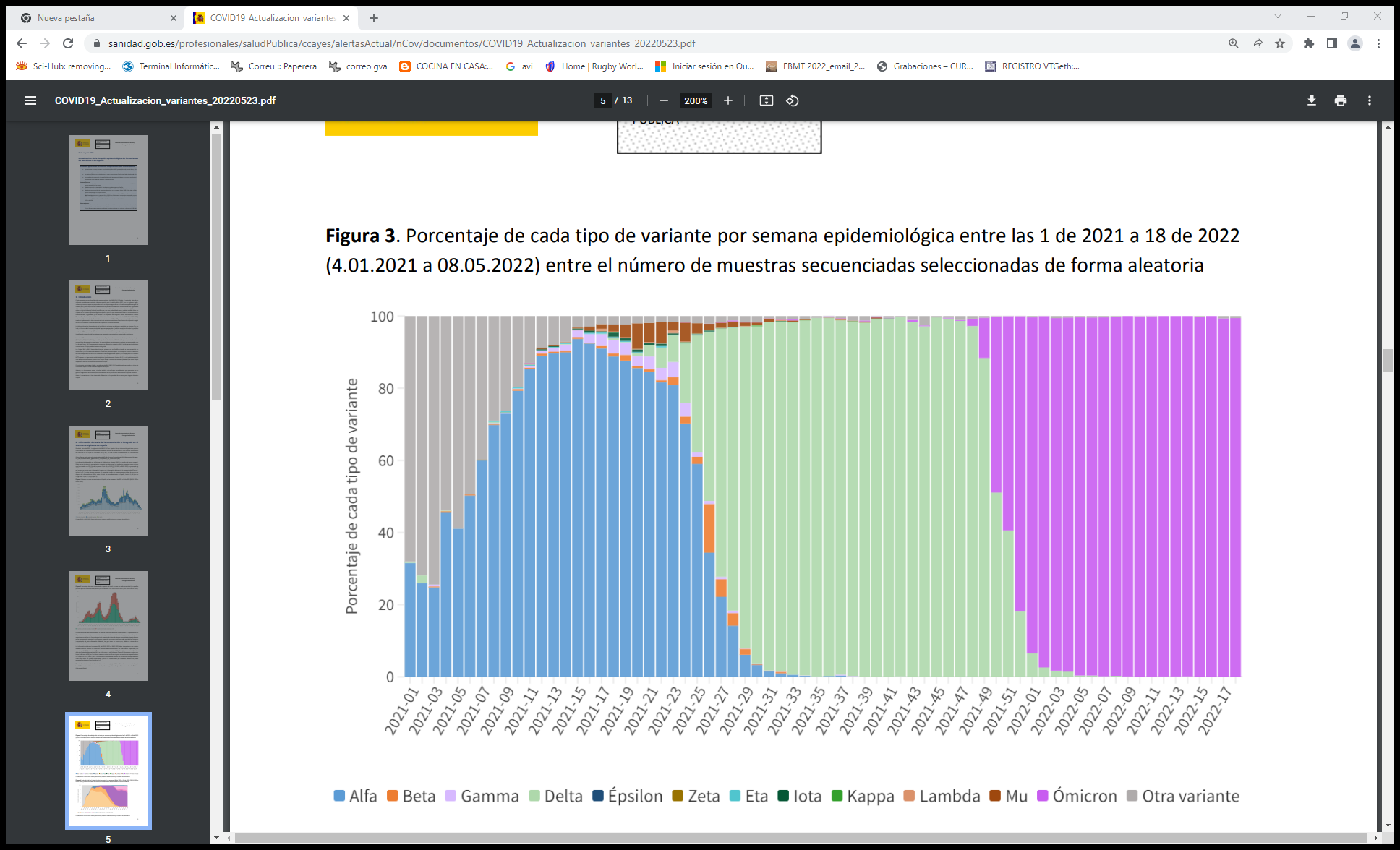


**Figure S2.** Spanish epidemiological curve of SARS-CoV-2 infection during the study period.

1. Available at site: [https://cnecovid.isciii.es/covid19/#evoluci%C3%B3n-pandemia](https://cnecovid.isciii.es/covid19/#evolución-pandemia).


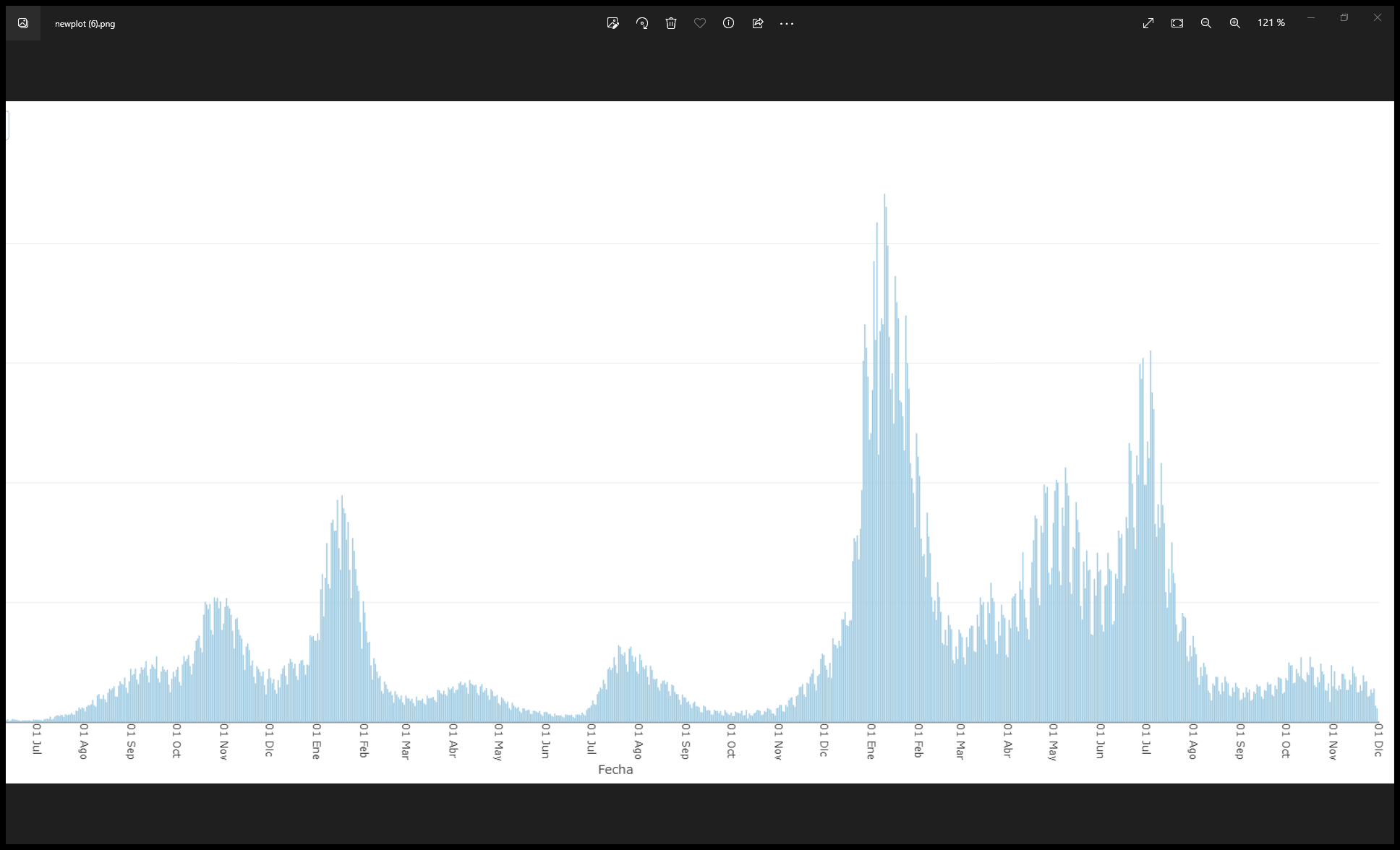

Supplement: Supplementary file 1 — supplementary file [file 41408_2022_778_MOESM1_ESM.docx]
